# The self-care dilemma of type 2 diabetic patients: The mechanism of self-regulation resource depletion

**DOI:** 10.1371/journal.pone.0208690

**Published:** 2018-12-06

**Authors:** Ligang Wang, Yan Yu, Ting Tao, Jingyi Zhang, Wenbin Gao

**Affiliations:** 1 CAS Key Laboratory of Mental Health, Institute of Psychology, Beijing, China; 2 University of Chinese Academy of Sciences, Beijing, China; University of Essex, UNITED KINGDOM

## Abstract

Self-care is important for type 2 diabetes mellitus (T2DM) patients’ disease prognosis, but there is a common phenomenon of self-regulation failure in T2DMs. In order to figure this problem out, the current study explored the interaction between self-regulation resource depletion and diabetes self-care based on the limited resource model of self-regulation. 104 patients were surveyed using the Self-Regulatory Fatigue Scale (SRF-S) and the Diabetes Self-care Scale (DSCS) in study 1. Study 2 recruited 30 T2DM patients and 30 healthy controls, and used a sequential-task paradigm to test the effect of self-regulation resource depletion on them. Participants in study 3 were 60 T2DM patients under different levels of self-regulation resource depletion manipulation, and their self-regulation performance was recorded and compared. Study 1 indicated that the correlation between self-regulation resource depletion and exercise and diet was significant and negative, suggesting that patients with greater self-regulation resource depletion performed poorly in exercise and diet. In Study 2, T2DM patients exhibited a poorer performance on the Spatial Incompatibility Task than the participants in the control group, suggesting that their self-regulation resource was insufficient. Study 3 indicated that there was no difference in Spatial Incompatibility Task performance, reaction time or error number among patients who were requested to complete a dietary record for one week and patients who were only requested to record eating times. This research demonstrated that low levels of diabetes self-care execution was associated with patients’ deficiency in self-regulatory resource, and self-care as a series of goal-directed behaviors consumed patients’ self-regulatory resources before these behaviors became a habit.

## Introduction

Type 2 diabetes mellitus (T2DM) is a long-term metabolic disorder that is characterized by high blood sugar, insulin resistance, and a relative lack of insulin. Long-term complications from high blood sugar include heart disease; stroke; diabetic retinopathy, which can result in blindness; kidney failure; and poor blood flow in the limbs, which may lead to amputation. Diabetes self-care, including diet, exercise, blood glucose monitoring, and insulin injection, is considered a cornerstone of diabetes mellitus treatment [1–3] and is particularly useful in reducing disease-related complications [4, 5]. Unfortunately, although health education regarding the essentiality and operational approach of diabetes self-care has become a regular treatment of T2DM patients, previous studies indicated that self-care was suboptimal in diabetes mellitus patients [6, 7].

Research from numerous theoretical perspectives has revealed the association between self-regulation and the regulation of health-related behavior [8, 9]. As described in detail previously [10], poor self-regulation has been linked to decreased compliance with behaviors such as exercise [11, 12], smoking cessation [13], and diet [12, 14]. Hagger et al. [15] observed that the capacity to regulate eating under self-regulation resource depletion conditions was reduced in individuals with high body mass indexes (BMI) who had frequently sought to reduce their food intake. This finding suggested that chronic self-regulation resource depletion impaired goal-directed diet behavior. Therefore, it is worth investigating whether self-care behaviors are associated with one’s level of self-regulation.

Muraven and Baumeister [16] developed a strength model of self-regulation, the basic tenet of which is that exercising self-regulation consumes people’s limited mental resources and that when these resources are exhausted, the capacity for further self-regulation is decreased. Generally, the strength model of self-regulation is tested by a “sequential-task” paradigm. In this paradigm, all participants are engaged in two consecutive tasks. For participants in the experimental group, both tasks require self-regulation, whereas for participants in the control group, only the second task requires self-regulation [17]. A large body of experimental data has consistently supported the aforementioned point [18].

Patients in chronic pain may suffer from chronic self-regulation resource depletion or self-regulatory fatigue. Solberg Nes, Carlson [19] conducted an experiment in which patients diagnosed with fibromyalgia or temporomandibular disorders and pain-free matched controls were exposed to an attention-control task followed by an anagram task. The results indicated that patients in the low self-regulation resource depletion condition displayed low levels of persistence, similar to patients and healthy controls in the high self-regulation resource depletion condition. In addition, for individuals experiencing co-morbid depression and chronic physical disease, their demands on the self-regulation system are compounded; that is, such patients not only need to assign a great deal of mental effort to disease management but also to emotion regulation, which leads to a rapid depletion of self-regulatory resource [20].

Health goal-directed behavior is an executive control process [21], and successful execution of goal-directed behaviors depends on a wide range of cognitive, affective, and motivational processes and effortful control [22]. Notably, researchers have observed that long-term demands on self-regulation may impair self-regulation [23, 24]. A new T2DM patient must change his/her lifestyle in many areas (e.g., diet, exercise, smoking, and drinking). Will execution of these new behaviors induce depletion of the patient’s self-regulation resources? In fact, Hofmann, Vohs [25] observed that the frequency and temporal closeness of attempts to resist desires affect people’s success in resisting subsequent desires on a given day.

The current research sought to provide a theoretical framework for the failure of diabetes self-care. Three studies were designed to test our hypotheses. In Study 1, using a survey, we tested the hypothesis that patients with self-regulatory fatigue or self-regulation resource depletion were more likely to perform poorly in the area of diabetes self-care. Study 2 sought to reveal whether T2DM patients were vulnerable to self-regulatory fatigue as a consequence of their chronic depleted condition. In this study, a “sequential-task” paradigm was used to measure the self-regulation resource depletion effect, and we proposed that compared with healthy group, participants with T2DM would perform more poorly on the second self-regulation task after self-regulatory effort. In Study 3, we explored whether recording one’s food intake for a week, a health goal-directed behavior, induced T2DM patients’ self-regulation depletion. Half of 60 T2DM patients were requested to conduct a difficult health goal-directed task for a week, whereas the other half executed an easy task. Both groups participated in a dependent measure of self-regulation after a week. We hypothesized that one week’s diet recording would induce T2DM patients’ self-regulation resource depletion, which made it difficult for them to execute self-control in the self-regulation task.

## Study 1

This study sought to examine the association between diabetes self-care and chronic self-regulation resource depletion.

### Methods

The study was approved by the Institutional Review Board of Institute of Psychology, Chinese Academy of Sciences (Approval number: H15003). All participants signed informed consent forms approved by IRB before participating in this experiment.

### Participants and procedure

A total of 104 T2DM patients (females = 33, mean age = 57.75±11.52) were recruited from a community hospital. Consistent with the World Health Organization, the definition of T2DM in the current study included a fasting plasma glucose≥7.0 mmol/l (126 mg/dl), or a plasma glucose≥11.1 mmol/l (200 mg/dl) two hours after the oral dose of 75g glucose load in the tolerance test, or a random plasma glucose ≥11.1 mmol/l (200 mg/dl) with typical hyperglycemia symptoms (e.g., polyuria, polydipsia, polyphagia, and weight loss).

Disease lasting for over 3 months, and having stable symptoms can be regarded as a chronic disease. At the beginning of disease diagnosis, patients may undergo a stage of disease deny, and can’t be in the patients’ role. In addition, patients have more negative emotions (such as anxious, depression, etc.) at this stage, which may influence their task performance. Therefore, in the current study, besides the diagnosis of T2DM, participants in the study met these criteria including: (1) having a course of T2DM for more than 3 months; (2) having clear awareness, can express accurately and having no communication barriers; (3) being voluntary to participate.

After informed of the purpose and requirements of this survey, all participants filled in the questionnaires on paper. When the survey questionnaires were completed, we offered them a gift worth RMB 20 Yuan (equivalently to 3.2 US dollars).

### Materials

In this study, Self-Regulatory Fatigue Scale (SRF-S) was used to measure participants’ levels of chronic self-regulation resource depletion. We translated and amended the scale developed by Nes, Ehlers, Whipple, and Vincent [26] to create a Chinese version [27]. The amended scale comprised 16 items and had 3 dimensions of cognition (e.g., I feel energetic), emotion (e.g., I tend to be upset) and behavior (e.g., I had impulse to break something) that could reflect one’s depleted condition in different aspects. It employed a Likert response format, with responses ranging from 1 (*not at all true*) to 5 (*very true*); higher scores reflected chronic self-regulation resource depletion or a scarcity of self-regulatory resources. The Cronbach’s alpha coefficient for the SRF-S was .84.

The Diabetes Self-care Scale (DSCS) comprised 26 items across six subscales: diet, exercise, glucose monitoring, taking medicine, foot care, and coping with hyperglycemia/hypoglycemia [28]. Each self-care behavior was rated on a 5-point Likert scale from 1 (*never done*) to 5 (*done very well*). According to Wang, Wan, and Shang’s [29] method, standard scores on six subscales were calculated using the following formula: Standard score = Original score/maximum score × 100. Higher scores indicated better performance on self-care. An individual’s performance was divided into three levels: poor (scores lower than 60), average (scores between 60 and 80), and good (scores higher than 80). In this sample, the Cronbach’s alpha coefficient for internal consistency was .94.

### Results

The means, SD, standard score, and bivariate correlations for self-regulation resource depletion and diabetes self-care are presented in Table 1. Patients performed well in taking medicine and poorly in monitoring glucose. The correlations between self-regulation resource depletion and exercise and diet were significant and negative, suggesting that patients with higher self-regulation resource depletion perform poorly in exercise and diet.

**Table 1:** Means, SD, standard score, and bivariate correlations for self-regulation resource depletion and diabetes self-care.

	M ± SD	Standard score	1	2	3	4	5	6
1 self-regulation resource depletion	45.50±8.20	—	—					
2 foot care	18.45±5.21	73.8	-0.138	—				
3 exercise	14.24±4.85	71.2	-0.269**	0.516**	—			
4 diet	22.65±5.64	75.5	-0.272**	0.591**	0.490**	—		
5 glucose monitoring	11.76±4.33	58.8	-0.113	0.553**	0.406**	0.484**	—	
6 taking medicine	13.79±2.09	91.9	-0.156	0.601**	0.435**	0.545**	0.285**	—
7 coping with hyperglycemia/hypoglycemia	15.83±3.76	79.2	-0.161	0.560**	0.446**	0.566**	0.621**	0.422**

Note.

**. p<0.01

### Discussion

Consistent with previous studies, our results indicated that self-care was suboptimal in diabetes mellitus patients in this study, with the exception of taking medicine. A survey of 200 T2DM patients by Liu and Huang [30] revealed that execution of self-care was unsatisfactory. A number of studies have consistently reported that diabetes self-care execution was imbalanced [31, 32]; specifically, patients scored higher in taking medicine and scored lower in glucose monitoring compared with the other self-care dimensions. Having experienced the effectiveness of medicine could explain good compliance with taking them. In addition, taking medicine is relatively independent of cognitive resource, while completing other aspects of self-care behaviors may expend more mental effort.

Study 1 indicated that there was significant association between self-regulatory fatigue and self-care behaviors. Specifically, patients with high self-regulatory fatigue performed poorly on diet and exercise.

Previous studies showed that self-regulation resource depletion would result in less effort execution, worse planning ability, and decreased persistence performance. A study of Wagner[33] used functional neuroimaging to test changes of chronic dieters’ brain activity while they were looking at desirable food, and found that participants under the depleted condition exhibited greater food cue-related activity in the orbitofrontal cortex. Martin Ginis and Bray [11] observed that participants exposed to a self-regulation resource depletion manipulation exhibited lower levels of work during a 10-minute bicycling task and tended to exert less effort in an ensuing exercise bout compared with control participants.

For T2DM patients, there are lots of things they should bear in mind and apply self-regulation resource in their daily lives, such as resisting the temptation of delicious food, doing exercise and so on. Also, T2DM patients can’t use plasma glucose effectively for the special property of the disease, which means there may be insufficient glucose supply when needed. Thus, they may undergo chronic self-regulation resource depletion, and can’t perform well on self-care behaviors that demand self-regulation. To investigate whether T2DM patients have experienced chronic self-regulation resource depletion and thus perform poorly on self-regulation task, study 2 compared the self-regulation ability after self-regulation resource depletion between T2DM patients and healthy individuals.

## Study 2

Study 2 explored whether T2DM patients were more likely to suffer from chronic self-regulation resource depletion and whether those patients exhibited poorer performance in executive functions than matched participants.

### Methods

The study was approved by the Institutional Review Board of Institute of Psychology, Chinese Academy of Sciences (Approval number: H15003). All participants signed informed consent forms approved by IRB before participating in this experiment.

### Participants and procedure

Thirty diagnosed T2DM patients were recruited from the department of endocrinology in a community hospital (females = 10, mean age = 52.7±10.5). The inclusion criteria were identical to those of study 1. And thirty healthy physical examination individuals at the same hospital were included as healthy controls (females = 10, mean age = 53.0±10.4). Participants with these characters were excluded in study 2: (1) co-morbid with severe neurological, renal, eye, heart, brain and foot complications; (2) having been diagnosed as T1DM; (3) having neurological disorders, Alzheimer’s disease or cognitive impairment; (4) having parachromatoblepsia, especially daltonism; (5) having been in similar studies before. When the number of participants reached 30 for each group, recruitment ceased. Participants of these two groups were matched by age (±2 years), gender, body mass index (BMI: ±2 points), and time of day for the experimental session (± 0.5 h).

In this experiment, two groups completed the tasks individually and were randomly assigned to one of two conditions (depleted or non-depleted). First, participants were requested to complete the Stroop Task to induce their depleted or non-depleted conditions, followed by a manipulation check. And then, all participants completed the Spatial Incompatibility Task.

### Materials

#### Stroop task

In this study, we used the Stroop Task to induce self-regulation resource depletion, consistent with previous studies [34, 35]. In the depleted condition, participants were requested to complete a typical Stroop task comprising trials in which the color words were incompatible with their ink colors (i.e., red in green or green in red) and to respond by pressing predefined keys on the keyboard (“Q” for green words and “P” for red words). There were 60 incongruent and 60 congruent trials, and their order was randomized. Twelve practice trials were designed to familiarize participants with the task. In the non-depleted condition, however, there were 120 congruent trials (i.e., red in red or blue in blue) in the task. Each trial began with a black fixation cross for 800 ms, followed by a target stimulus for a maximum of 2000 ms. Stroop task took approximately 5 minutes. And after that, a manipulation check was carried out, to gauge the task difficulty, participants’ tension level induced by the task, and the effort extent to which participants were making while performing the task, using a 1 (*strongly disagree*) to 5 (*strongly agree*) scale.

#### Spatial Incompatibility Task

**Fig 1:**
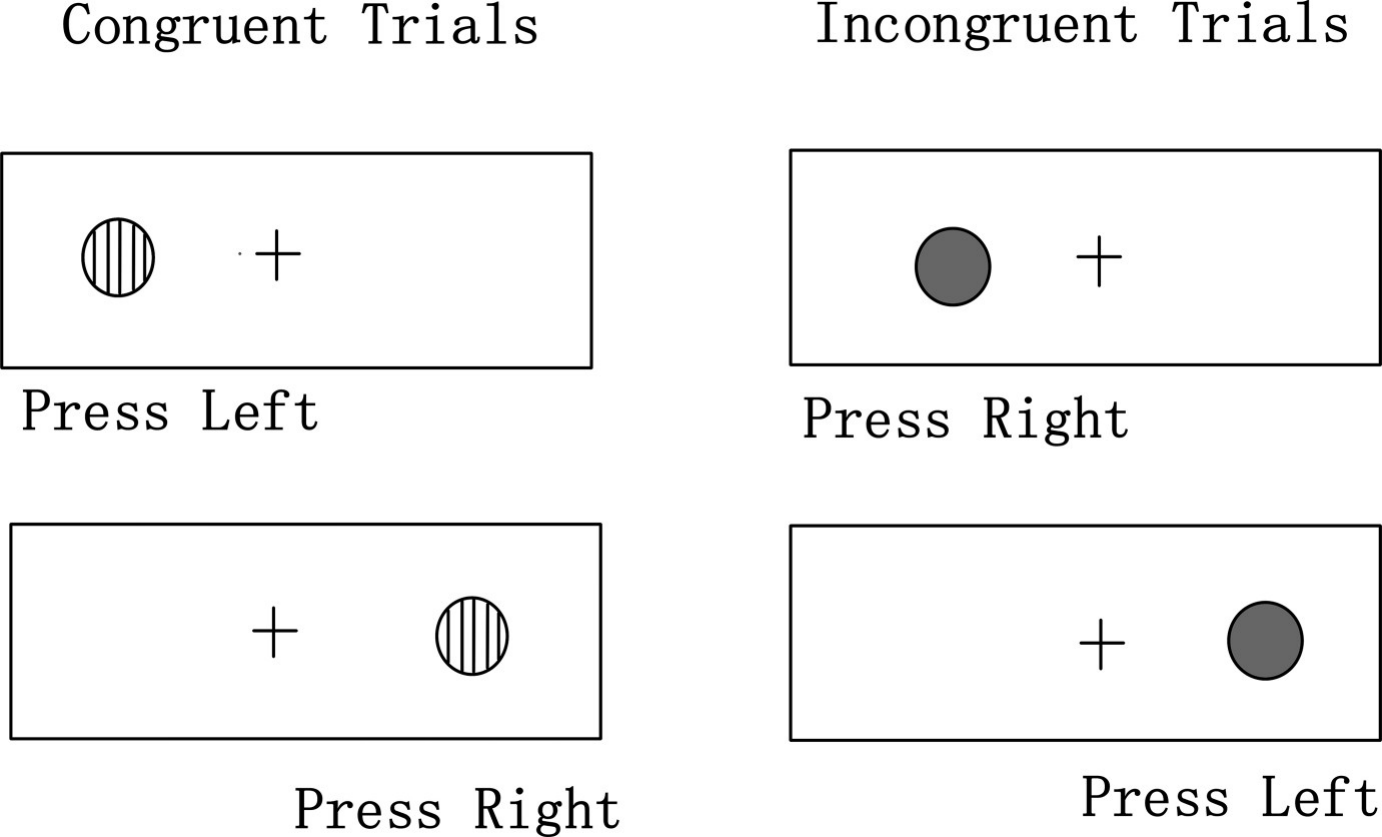
Spatial Incompatibility Task.

The Spatial Incompatibility Task was used to assess executive functions such as attention and cognitive control[36, 37]. In this task, participants saw pictures of either a striped or a gray dot presented on the left or right side of a fixed cross (see Fig 1). If participants saw a striped dot, they should press a key on the same side as the dot as quickly as possible; conversely, when a gray dot appeared, they should press a key on the opposite side. The ‘Q’ key was defined as the left key, and the ‘P’ key was the right key. Thus, trials in which a striped dot appeared were “congruent” trials, and the others were “incongruent” trials. After 20 practice trials, 40 incongruent and 40 congruent test trials were carried out, and their order was randomized. Each trial began with a fixation cross presented for 2000 ms; followed by a 200 ms presentation of the striped or gray dot, and then a blank respond screen lasting for 4000 ms.

### Results

Although no significant difference existed in the tension induced by the task in the two groups (depleted: M = 2.41, SD = 1.13; non-depleted: M = 2.23, SD = 0.99) (t[59] = 0.47, *p* = 0.63, Cohen’s *d* = 0.17), depleted participants indicated that the Stroop task was more difficult (depleted: M = 2.29, SD = 0.89; non-depleted: M = 1.49, SD = 0.65) (t[59] = 4.54, *p*<0.01, Cohen’s *d* = 1.03) and reported making an effort (M = 3.06, SD = 1.08) more often than non-depleted participants (M = 2.24, SD = 0.83) (t[59] = 3.67, *p*<0.01, Cohen’s *d* = 0.93). Thus, we can be reasonably confident that we successfully manipulated self-regulation resource depletion across groups.

A 2 (depleted, non-depleted) × 2 (T2DM, non-T2DM) ANOVA on the Spatial Incompatibility Task performance was conducted, and the results are presented in Table 2. In the space-compatible condition, with regard to reaction time (RT), neither a main effect nor an interaction between the depletion and subject type were significant; as for error number, there was a significant main effect of subject type; however, neither a main effect for the depletion condition nor an interaction between the depletion and subject type were significant. In the space-incompatible condition, main effects of subject type on both RT and error number were significant. However, the effect of self-regulation resource depletion and subject type-by-condition interaction was not observed. Although the depleted group exhibited a poorer performance in the Spatial Incompatibility Task than the non-depleted group, the difference was not statistically significant. Our results indicated that, compared with the matched group, T2DM participants performed poorly in the Spatial Incompatibility Task, suggesting that their self-regulation resources were insufficient.

**Table 2:** Primary effects and interactive effects between self-regulation resource depletion and Participants type in the Spatial Incompatibility Task.

	Compatible condition						Incompatible condition					
	Reaction time (ms)			Error number			Reaction time (ms)			Error number		
	*M ± SD*	*F*	*η*^*2*^	*M± SD*	*F*	*η*^*2*^	*M ± SD*	*F*	*η*^*2*^	*M ± SD*	*F*	*η*^*2*^
Self-regulation resource depletion												
Depleted group	685.61±103.93	1.62	.01	7.53±6.14	.82	.01	680.40±109.12	.81	.01	7.77±8.02	0.23	.01
Non-depleted group	650.90±103.93			6.30±4.94			655.50±109.28			6.80±8.70		
Participants Type												
T2DM group	673.33±83.54	.14	.03	8.73±6.09	7.13**	.11	691.83±107.75	5.96*	.07	10.60±10.10	10.89**	.16
Non-T2DM group	663.18±123.27			5.10±4.35			664.07±106.69			4.97±3.97		
Self-regulation resource depletion× Participants Type		.10	.01		1.56	.03		.98	.02		0.10	.01

Note.

*p<0.05

**p<0.01

### Discussion

Study 2 used a “sequential-task” paradigm to examine whether T2DM patients were more likely to suffer from chronic self-regulation resource depletion and whether these patients perform more poorly on executive functions. The hypotheses of this study were partially supported. T2DM patients performed poorly on the Spatial Incompatibility Task, suggesting that their executive functions were impaired.

This result was consistent with the reports of previous studies[38, 39]. For example,van den Berg, Reijmer [40] observed that, compared with controls, patients with T2DM exhibited a moderate decay in information-processing speed and executive functions. In a brain-imaging study, de Bresser, Tiehuis [41] reported that the total brain volume of patients with type 2 diabetes was significantly smaller than control participants’. Biessels and Reijmer [42] indicated that the cognitive dysfunction observed in T2DM patients may stem from global brain atrophy and the increased burden of small-vessel disease.

Unexpectedly, the effect of self-regulation resource depletion was not observed in this study. Failure to detect self-regulatory fatigue maybe associated with the fact that the majority of participants were elder adults. A previous study observed that only younger participants (<25 years) were susceptible to depletion effects, because their incomplete development of the prefrontal cortex, while in an older group (40–65 years), the effect of depletion disappeared[43].

Study 2 found that T2DM patients showed worse performance on executive functions when compared with healthy controls, thus indicated that T2DM patients were more susceptible to self-regulation resource depletion. But the reason why they were likely to be influenced by depleted condition was still ambiguous. To investigate the causality between T2DM patients’ chronic self-regulation resource depletion and goal-directed self-care behaviors, study 3 was carried out.

## Study 3

Study 3 tested whether a health goal-directed behavior, recording one’s diet for one week, would induce T2DM patients’ self-regulation resource depletion.

### Methods

The study was approved by the Institutional Review Board of Institute of Psychology, Chinese Academy of Sciences (Approval number: H15003). All participants signed informed consent forms approved by IRB before participating in this experiment.

### Participants and procedure

Sixty patients with T2DM participated in Study 3, the inclusion and exclusion criteria were identical to the criteria of Study 2. When the number of participants reached 30 for each group, recruitment ceased. Thirty patients (females = 9, mean age = 56.1±9.7) were randomly assigned to the health goal-directed group and the other participants (females = 9, mean age = 52.5±10.9) to the control group. In the health goal-directed group, participants were requested to record what they ate every day during the following week. In the control group, however, participants only recorded when they had a meal every day during the following week.

In this study, patients were requested to complete a diet recording task for one week, and then finished a manipulation check, after that, all participants completed the Spatial Incompatibility Task.

### Materials

#### Diet recording task

**Fig 2:**
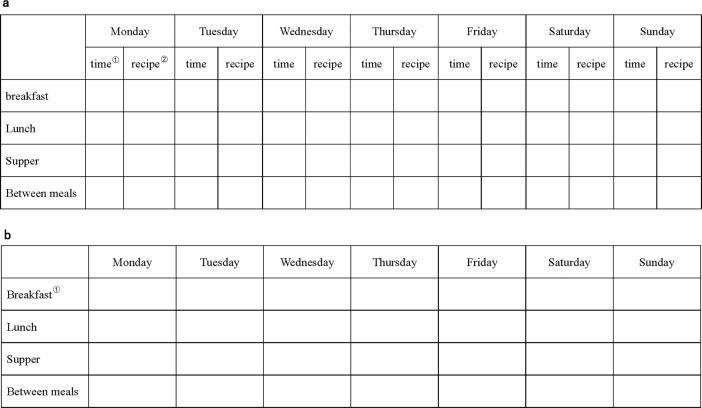
A diet record chart for a week. Chart a was designed for the health goal-directed group, while Chart b was used by control group. NOTE. ① Please provide a time interval, for example, 7:00 ~ 7:30. ②. Please provide the food information in as much detail as possible, for example, 250 ml milk, one egg, 150 g rice.

The health goal-directed group were provided with a diet recording chart (see Fig 2A) to record everything they ate for a week whereas the control group used the chart presented in Fig 2B to record their mealtimes for the next 7 days. In addition, all participants reported every day whether they wanted to continue the recording task.

#### Spatial Incompatibility Task

The experimental manipulation conducted in Study 3 was identical to the manipulation in Study 2. In addition, we defined the Spatial Incompatibility Task performance as dependent measures.

### Results

Inconsistent with the hypothesis, no group difference was observed in the Spatial Incompatibility Task performance, neither on RT (Reaction Time) nor error number (see Table 3).

**Table 3:** Main effects of self-regulation resource depletion on Spatial Incompatibility Task performance.

	Compatible condition						Incompatible condition					
	Reaction time (ms)			Error number			Reaction time (ms)			Error number		
	*M ± SD*	*t*	*d*	*M ± SD*	*t*	*d*	*M ± SD*	*t*	*d*	*M ± SD*	*t*	*d*
Health goal-directed group	682.55±73.50	1.21	.31	7.70±6.84	1.44	.37	672.58±89.79	.86	.22	6.50±8.62	.25	.06
Control group	657.80±84.46			5.53±4.57				651.75±98.21			6.00±7.11		

**Fig 3:**
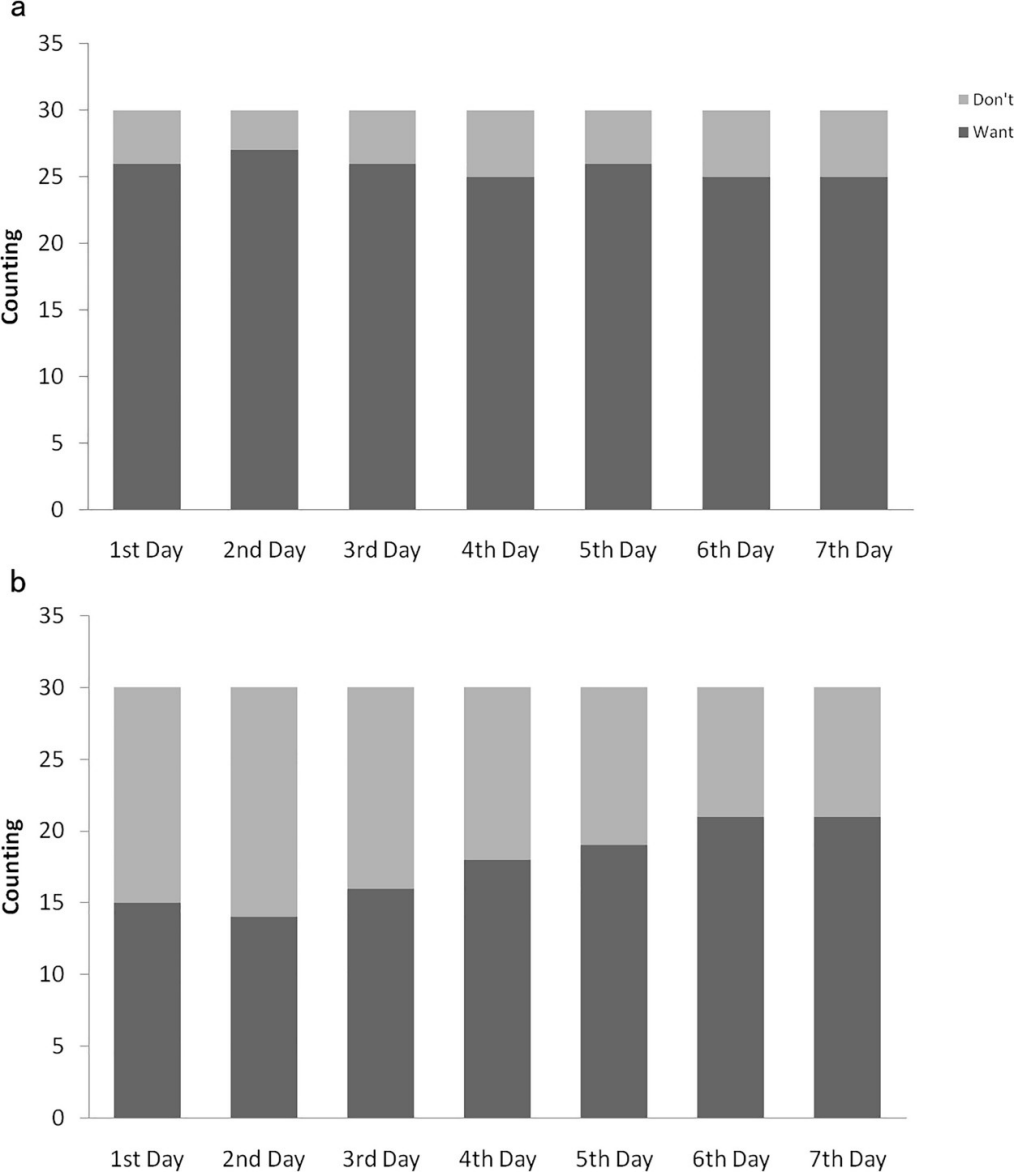
Willingness to continue the recording task. Plate a displays results of the health goal-directed group, and plate b represents control group.

We compared the participants willingness to continue the recording task between the two groups (see Fig 3). Chi-square tests indicated that in the first three days, the health goal-directed group reported less willingness to continue the task than the control group (the first day: χ^2^ = 3.89, *p* = 0.049; the second day: χ^2^ = 3.96, *p* = 0.042; the third day: χ^2^ = 3.12, *p* = 0.074); while in the remaining days, the two groups’ willingness reached unanimity (the fourth day: χ^2^ = 2.87, *p* = 0.109; the fifth day: χ^2^ = 2.50, *p* = 0.119; the sixth day: χ^2^ = 1.01, *p* = 0.297; the seventh day: χ^2^ = 1.01, *p* = 0.297).

### Discussion

In study 3, we did not observe a significant difference in Spatial Incompatibility Task performance between the health goal-directed group and the control group. An explanation might be that adapting to the health goal-directed task negated the self-regulation resource depletion effect. In an experimental study, Dang, Dewitte [35] designed an elegant adaptation condition in addition to two conditions generally used in studies examining the depletion effect (i.e., the depletion condition and the control condition). In the adaptation condition, participants were requested to complete two more blocks of the Stroop task than the participants in another two conditions. The results indicated that once people had adapted to the demands of the initial task, their performance on the subsequent self-regulatory tasks would be free from impairment. Similarly, participants in this study might have adapted to the demands of diet recording, which was supported by patients’ self-reported data. Specifically, the health goal-directed group reported less willingness to continue the task than the control group only during the first three days.

In addition, a wealth of studies demonstrated that self-regulatory capacity was improved significantly by repeated exercises of self-regulation, such as a hand-grip exercise task[44] and a non-dominant-hand task[45]. Which might also account for the finding in study 3 that patients showed no significant depleted effect after one week’s diet recording task.

The finding in study 3 didn’t detect the aftereffect on self-regulation induced by one week’s diet recording task. But from the difference on participants’ willingness to continue the recording task, it could be inferred that patients might have experienced a quick self-regulatory process while completing self-care behaviors, which indicated that at the beginning of developing a goal-directed behavior, people might suffer from self-regulation resource depletion, but when the behavior had become a habit, the depletion effect would disappear.

## General discussion

The current study intended to give an explanation for T2DM patients’ self-care dilemma from the perspective of self-regulation resource depletion. We assumed that low execution of diabetes self-care was associated with patients’ deficiency in self-regulatory resources; it was also hypothesized that self-care as a series of goal-directed behaviors consumed patients’ self-regulatory resources before these behaviors became a habit. In a previous study, Wang, Tao, Fan, Gao, and Wei [10] observed that people with high levels of chronic self-regulation resource depletion were less successful in goal adherence than people with less chronic self-regulation resource depletion. Once more, this idea was supported in patients with T2DM. Furthermore, Study 2 indicated that T2DM participants exhibited poorer executive function. These findings lead to the following recommendations: First, before guiding diabetes self-care, clinical staff should estimate the level of self-regulation of T2DM patients. An easy or step-by-step approach may be appropriate for patients with chronic self-regulation resource depletion. Second, more support should be provided to patients when patients are executing a difficult self-care behavior such as exercise, diet adjustment, or smoking cessation. Although the hypothesis that self-care consumed patients’ self-regulatory resources was not supported in this study, we observed that patients would adapt to the demands of self-care and that this adaption might cancel the self-regulation resource depletion effect. Therefore, our third recommendation is that, in the early stages of health management, clinical staff should provide additional supervision to patients and assure their patients that the difficulty of self-care will decrease when self-care becomes a habit.

This research makes several noteworthy contributions to the research of self-regulation resource depletion. On an extremely general level, this study provides new data to understand the relation between self-regulation resource depletion and goal adherence of T2DM patients, as well as demonstrating their low executive functions. Furthermore, the application of a multi-method approach improves the reliability of data. However, the findings of this study must be viewed while considering several limitations. First, although the course of the disease was matched between experimental and control groups in this study, it varied greatly from individual to individual. Future research should recruit more newly diagnosed T2DM patients. Second, there was only one behavior designed to test the effect of self-regulation resource depletion in the current study, which failed to represent the real situation of T2DM patients. Studies in the future can include more behavioral data in real life and improve the ecological validity.

In conclusion, this research examined the interaction between self-regulation resource depletion and diabetes self-care behaviors. The results can be summarized as follows: (1) there was a negative correlation between chronic self-regulation resource depletion and self-care behaviors; (2) T2DM patients showed more susceptibility to self-regulation resource depletion and performed poorer on self-regulation task.

The current study demonstrated that the execution of self-care behaviors needed the expenditure of self-regulation resource, and for T2DM patients, who had been under condition of chronic self-regulation resource depletion, the formation of health goal-directed behaviors should be attached great importance to, especially at the beginning stage of habit changing.
